# Exosomes play an important role in the process of psoralen reverse multidrug resistance of breast cancer

**DOI:** 10.1186/s13046-016-0468-y

**Published:** 2016-12-01

**Authors:** Xiaohong Wang, Chengfeng Xu, Yitong Hua, Leitao Sun, Kai Cheng, Zhongming Jia, Yong Han, Jianli Dong, Yuzhen Cui, Zhenlin Yang

**Affiliations:** 1 Department of Thyroid and Breast Surgery, The Affiliated Hospital of Binzhou Medical University, 522 Yellow Three Road, Binzhou, Shandong 256603 People’s Republic of China; 2 Department of Nneurosurgery, The Affiliated Hospital of Binzhou Medical University, Binzhou, Shandong 256603 People’s Republic of China

**Keywords:** Breast cancer, Chemoresistance, Exosome, Psoralen

## Abstract

**Background:**

Release of exosomes have been shown to play critical roles in drug resistance by delivering cargo. Targeting the transfer of exosomes from resistant cells to sensitive cells may be an approach to overcome some cases of drug resistance.

**Method:**

In this study, we investigated the potential role of exosomes in the process of psoralen reverse multidrug resistance of MCF-7/ADR cells. Exosomes were isolated by differential centrifugation of culture media from MCF-7/ADR cells (ADR/exo) and MCF-7 parental cells (S/exo). Exosomes were characterized by morphology, exosomal markers and size distribution. The ability of ADR/exo to transfer multidrug resistance was assessed by MTT and real-time quantitative PCR. The different formation and secretion of exosomes were detected by immunofluorescence and transmission electron microscopy. Then we performed comparative transcriptomic analysis using RNA-Seq technology and real-time quantitative PCR to better understand the gene expression regulation in exosmes formation and release after psoralen treatment.

**Results:**

Our data showed that exosomes derived from MCF-7/ADR cells were able to promote active sequestration of drugs and could induce a drug resistance phenotype by transferring drug-resistance-related gene MDR-1 and P-glycoprotein protein. Psoralen could reduce the formation and secretion of exosomes to overcome drug resistance. There were 21 differentially expressed genes. Gene ontology (GO) pathway analysis and Kyoto Encyclopedia of Genes and Genomes (KEGG) pathway analysis showed that the most significantly expressed genes were linked to PPAR and P53 signaling pathways which were related to exosomes formation, secretion and cargo sorting.

**Conclusions:**

Psoralen can affect the exosomes and induce the reduction of resistance transmission via exosomes might through PPAR and P53 signaling pathways, which might provide a novel strategy for breast cancer resistance to chemotherapy in the future.

## Background

Chemotherapy plays an important role against breast cancer, but its development is mainly restricted by drug resistance. Breast cancer cells effectively evade chemotherapy by a number of different processes and strategies. Besides inherent resistance, acquired drug resistance has become common. The mechanisms of acquired drug resistance are under intense research, and growing attention has been paid to the the transfer of exosomes as mediators of intercellular communication from a drug-resistant to a drug sensitive cancer cell.

Exosomes are small (30–150 nm) membrane vesicles that originate from the endosomal membrane compartment [1] that are released upon fusion of multivesicular bodies with plasma membranes from diverse cell types. Strikingly, resistance transmission is also one such role, through their ability to internalize into surrounding cells or distant tissues and the constant transfer of proteins, bioactive lipids, messenger RNAs (mRNAs), and microRNAs (miRNAs) [2–5]. Recently, it has been demonstrated that the release of exosomes maybe a mechanism in drug resistance in cancer cells by transferring drug transporter nucleic acid and proteins and/or accumulating anticancer drugs [6]. Such phenomenon was observed in several tumour models, including ovarian cancer [7], prostate cancer [8, 9] and osteosarcoma [10]. In colon cancer cells, enhanced secretion of miR-145 and miR-34a via exosomes increased the cells’ resistance to 5-fluorouracil [11]. Docetaxel resistance is related to the enhancement of exosome secretion in a prostate cancer model, probably due to docetaxel efflux through exosomes [12]. Therefore, reducing formation and secretion of exosomes may be a novel therapeutic strategy for adjuvant cancer treatment by restoring drug sensitivity in breast cancer [4, 13, 14].

In previous study, we have confirmed that psoralen could reverse MDR in human breast cancer MCF-7/ADR cells. We suggest that in breast cancer, psoralen probably acts through reducing the formation and secretion of exosomes and that this mechanism may contribute to the multidrug resistance (MDR) reversal effect. In this study, we analyzed the effect of exosomes in transmitting drug resistance and confirmed the role of psoralen in reversion of multidrug resistance via exosomes providing new insights for overcoming drug resistance.

## Methods

### Cell lines

MCF-7 and MCF-7/ADR cells (Nanjing KeyGen Biological Technology Development Co. Ltd, Nanjing, China).

### Cell culture

MCF-7 and MCF-7/ADR cellswere maintained in RPMI-1640 medium supplemented with 10% fetal bovine serum, 100 U/ml penicillin G and 100 mg/ml streptomycin in ahumidified atmosphere of 5% CO_2_ at 37 °C. MCF-7/ADR were cultured in the medium containing 1ug/ml ADR in order to maintain the MDR phenotype, and were then maintained in drug-free medium for at least two days prior to use. The culturemedium was changed for every 2 days.

### Exosomes isolation

To reduce the influence of exosomes in FBS, FBS was depleted of exosomes by ultracentrifugation at 200,000 g at 4 °C for 16 h, supernatants were filtered through a 0.22 mm sterile filter and subsequently mixed with serum free media to prepare exosome depleted cell culture media containing 10% FBS. Cells were grown in exosome depleted culture media up to 70% confluence. To harvest exosomes, 200 ml cell culture media respectively from MCF-7, MCF-7/ADR cells were collected and cleared of debris and non-exosome vesicles by sequential centrifugations (200 g for 10 min, 2000 g for 20 min, 10,000 g for 30 min) and then centrifuged at 100,000 g and 4 °C for 70 min to pellet exosomes (Avanti J-30I, Beckman Coulter, USA) [15]. The final pellets were used directly or resuspended in PBS or SDS sample buffer and stored at −80 °C for follow-up test. The exosomes extracted from the supernatant of MCF-7 and MCF-7/ADR cells were named S/exo and ADR/exo, respectively.

### Identification and characterization of exosomes

Exosomes were analyzed by transmission electron microscopy using negative staining. A drop of exosomes (about 10 μL) was added on copper grid for 1 min, dried at 65 °C and observed on a HT7700 transmission electron microscope (HITACHI, Japan) equipped and operated at an acceleration voltage of 80 kV. Images were taken using a Gatan CCD (Gatan, Inc., US). Exosome purity was assessed by western blot analysis. Total cellular and exosomal proteins were respectively extracted from cells and exosomes using SDS lysis buffer (250 nM Tris-HCl, pH 7.4, 2.5% SDS). Proteins (10 mg/mL) were separated on 10% SDS-PAGE gels and transferred to a PVDF membrane. The antibodies used for CD63, TSG101, calnexin and P-gp (Proteintech Group, CHI, USA), and enhanced chemiluminescence (ECL) plus kit (Millipore, America) was applied for visualization. The size distribution was detected by a nano-ZS90 analyzer (Malvern, Worcestershire, UK) after diluted 10 times.

### PKH67-labeled exosomes absorbed by MCF-7 cells

Exosomes were labeled with PKH67 (Sigma-Aldrich, USA) according to the manufacturer’s recommendation [9]. Briefly, while the isolated exosomes from 200 ml culture media were resuspended in 500 μL Diluent C, 4 μL PKH67 was diluted in another 500 μL Diluent C. Then, these two solutions were mixed gently for 5 min, after which 5 ml 1% bovine serum albumin was added to bind the excess dye. The mixture was subsequently ultracentrifuged at 100,000 g for 2 h at 4 °C, washed with PBS by ultracentrifugation and finally resuspended in complete medium. As the negative control, exosomes without PKH67 staining were prepared. Incorporation of exosomes into MCF-7 was visualized by fluorescence microscopy after incubation with PKH67-labeled S/exo and ADR/exo for 30 min at 37 °C. 24 h later, they were observed under confocal laser scanning microscope. The analysis of the intracellular distribution of adriamycin was carried out by taking advantage of the inherent fluorescence of the drug.

### Co-culture assays

To assess the effect of exosomes on the potential transmission of drug-resistance, cells were seeded in 6-well plates (2.5 × 10^5^cells/well). After cells had attached, the media were removed and fresh media containing exosomes quantitated by the Bicinchoninic acid (BCA) assay [16, 17]. 50 mg/ml ADR/exo, S/exo or PBS (control) were added. The expression of drug-resistance-related gene MDR-1, MRP and LRP were assessed by qRT-PCR following 24 h incubation. Total RNA from cells (*n* = 3) was extracted using TRIzol reagent (Invitrogen, Life Technologies, Monza, Italy) and was reverse transcribed using the MULV Reverse Transcriptase kit (Applied Biosystems, Thermo Fisher Scientific). The primer sequences were designed and supplied from Sangon Biotech Co., Ltd. (Shanghai, China) as follows: MDR1, F 5′-CCCATCATTGCAATAGCAGG-3′ and R 5′-GTTCAAACTTCTGCTCCTAG-3′; LRP, F 5′-GTCTTCGGGCCTGAGCTGGTGTCG-3′ and R 5′-CTTGGCCGTCTCTTGGGGGTCCTT-3′; MRP, F 5′-TCTCTCCCGACATGACCGAGG-3′ and R 5′-CCAGGAATATGCCCCGACTTC-3′; β-actin, F 5′-TGTCACCAACTGGGACGATA-3′ and R 5′-GGGGTGTTGAAGGTCTCAAA-3′. The cDNA (1 μl) was amplified by PCR on a CFX96 Touch Real-Time PCR Detection System (Bio-Rad Laboratories, Inc., Hercules, CA, USA), at 95 °C for 1 min and 45 s, followed by 35 cycles of 95 °C for 30 s and 60 °C for 30 s, with a final extension at72°C for 7 min. The data were analyzed by 2^-ΔΔCq^ method.

### MTT assay

The concentration of adriamycin inhibiting 50% of MCF-7 and MCF-7 cells incubated with ADR/exo (IC50) were analyzed by MTT assay. Briefly, MCF-7 and MCF-7 cells incubated with ADR/exo were seeded at a cell density of 8 × 10^3^ cells per well (in triplicate) in 96-well plates. After 24 h, they were treated with adriamycin at different concentration for 48 h. After the treatment, cells were incubated with 10 μL MTT for 4 h, and then discarded the medium and added 200 μL DMSO. The spectrophotometric absorbance was measured at 490 nm with enzyme-labeling instrument after the crystals were fully dissolved. The IC50 was calculated on SPSS 16.0 (SPSS Inc., Chicago, USA).

### Adriamycin accumulation assay

The distribution of adriamycin in MCF-7 and MCF-7 cells incubated with ADR/exo was determined using a confocal laser scanning microscope. The cells on confocal dishes were treated with 5 μg/ml adriamycin for 4 h and then examined. Adriamycin fluorescence was determined with excitation at 488 nm using an argon laser and the emission was collected through a 530-nm long-pass filter.

### Adriamycin are detected in released exosomes

The isolated exosomes were lysed in CelLytic™M buffer [18]. We used a UV spectrophotometer (GeneQuant1300, GE Healthcare, USA) to analyze adriamycin in the released exosomes of MCF-7/ADR and MCF-7/ADR cells treated with 5 μg/ml adriamycin for 4 h. The adriamycin maximum absorption wavelength was at 254 nm. We set the standard curve by gradient concentration. The equal volume Phenol Red-free RPMI-1640 medium as blank control. The absorbance value (A) was used to evaluate the relative concentration of adriamycin associated with exosomes.

### Different amounts of formation and secretion of exosomes

To evaluate the roles of exosomes in resistance transfer, the amounts of formation and secretion of exosomes were analysed by confocal microscopy (FITC - labeled CD63) and Scanning electron microscopy in MCF-7, MCF-7/ADR and MCF-7/ADR + psoralen cells. Cells were washed twice with PBS and fixed in 2.0% glutaraldehyde in 0.1 M phosphate buffer, then post-fixed in 1% osmium tetroxide buffer. After dehydration in a graded series of ethanol, cells were embedded in spur resin. Thin sections (70 nm) were cut on an ultramicrotome. The sectioned grids were stained with saturated solutions of uranyl acetate and lead citrate. The sections were examined under electron microscope.

### Preparation of a cDNA library for RNA-seq

Total RNA were respectively extracted from cells of MCF-7, MCF-7 + psoralen, MCF-7/ADR and MCF-7/ADR + psoralen at 24 h using TRIzol reagent. ND-1000 Nanodrop and Agilent 2200 TapeStation were used to explore the sample quality. RNA libraries were then generated using the NEBNext® Poly(A) mRNA Magnetic Isolation Module from Illumina (San Diego, CA, USA) according to the manufacturer’s instructions. The cDNA fragments were sequenced in paired end lane for 101 cycles using the Illumina HiSeq3000.

### Analysis of RNA-seq data

Raw sequence files underwent a quality control analysis using FastQC (version 0.10.1, http://www.bioinformatics.babraham.ac.uk/projects/fastqc/). To avoid low-quality data, we clipped and trimmed the reads using the FASTXToolkit (version 0.0.14, http://hannonlab.cshl.edu/fastx_toolkit/). For the analysis of differentially expressed genes, the quality-checked reads for each sample were processed using TopHat (version 2.0.10) software based on the Homo sapiens NCBI hg19 reference genome sequence. The differential gene-expression values for each sample were calculated by DEseq based on the RPKM (reads per kilo bases per million reads method) and further verified by real-time quantitative PCR (RT-qPCR) (Table 1). A heat map was generated with the differential expression genes by Guangzhou RiboBio Co., Ltd.. The acquired data were deposited in the Gene Expression Omnibus database (accession number: PRJNA274725). KEGG (http://www.genome.jp/kegg/) was conducted to determine the most significant canonical pathways in the data sets.

**Table 1:** List of primers used for candidate genes

Gene name	Forward primer (5'to 3')	Reverse primer (5'to 3')	prodSize	Tm (°C)
KRTAP2-3	CTCCACCTTGTCCTCCCTGA	GGCAGGGCTCGCAGATG	155	61
FOSL1	GAAGGCCTTGTGAACAGGAG	CTTCCAGCACCAGCTCTAGG	111	60
MMP1	CCAAGGTCTCTGAGGGTCAA	CTGGTTGAAAAGCATGAGCA	114	60
MYPN	AATGAGACCATCCCTTGCAC	TGGCTGACAACGTGTACCAT	121	60
ENSG00000231826.1	CCAGCTTTGTCCTGACCTTC	GTTGCTGCCAAACTGCTACA	121	60
ALPP	AGAATCTGGTGCAGGAATGG	AGGCTCAAAGAGACCCATGA	122	60
ENSG00000237862.1	CATGGCCTCCTGATGAAGAT	AAGCAGGTTGGAAGTCAGGA	119	60
LRRC15	TCGACCACCTGGTAGGACTC	TTCTCATACAGCCGGAGGAC	115	60
TMEM40	AGATGGGAAGGCTGGACTCT	TTGCTCTCTGAGGAGGAGGA	120	60
ALPL	GACAAGAAGCCCTTCACTGC	AGACTGCGCCTGGTAGTTGT	120	60
TMEM200A	GCTATGGCCGTTCTTGGATA	AAAGAAGCGAACCACCACAC	120	60
LCE2A	CAGCAAAACCAGCAGCAGT	CCACAGCAGGAAGAGACTGG	125	61
SIRPB1	CTGTGCTATGACGTCCCTGA	GTTGTTACCCGTGGGAAGTG	114	60
SESN3	ATGCTTTGGCAAGCTTTGTT	GCAAGATCACAAACGCAGAA	112	60
PEAR1	CCTGATCCATGACCGAGACT	CCGTACTGGAGGCAAGTCAT	124	60
HSD17B6	TCTGGGGACTGGTGAACAAT	AAGGTCACCTGGATCACACC	121	60
ENSG00000261159.1	GGCTGCATTTTGTCCTGTCT	TGACTGTGCTGACCAAGAGC	127	60
INHBA	AGACGCTGCACTTCGAGATT	GGATGGTGACTTTGGTCCTG	120	60
ARNT2	GGATGAGGTGTGGAAATGCT	ACCACAGCATATTGGGCTTC	121	60
IGFN1	GGGTCAGAACAGCCTATGGA	CATCTGCTGATCCCATTCCT	120	60
RXFP4	TGCCTGTCAAATTCCTAGCC	CTCTCCGGGCACAGTTACTC	120	60

The table displays the List of primers used for candidate genes after psoralen treatment. It holds the primers sequences, production size and the annealing temperature (Tm)

### Statistical analysis

All experiments were performed in triplicate, and representative data were shown from three separate experiments. A statistical analysis was performed using a *t*-test or one-way ANOVA using the SPSS 17.0 statistical software. All experiments were performed in triplicate, and *p* < 0.05 was considered statistically significant. GraphPad was used for graph generation.

## Results

### Identification and characterization of exosomes

**Fig. 1:**
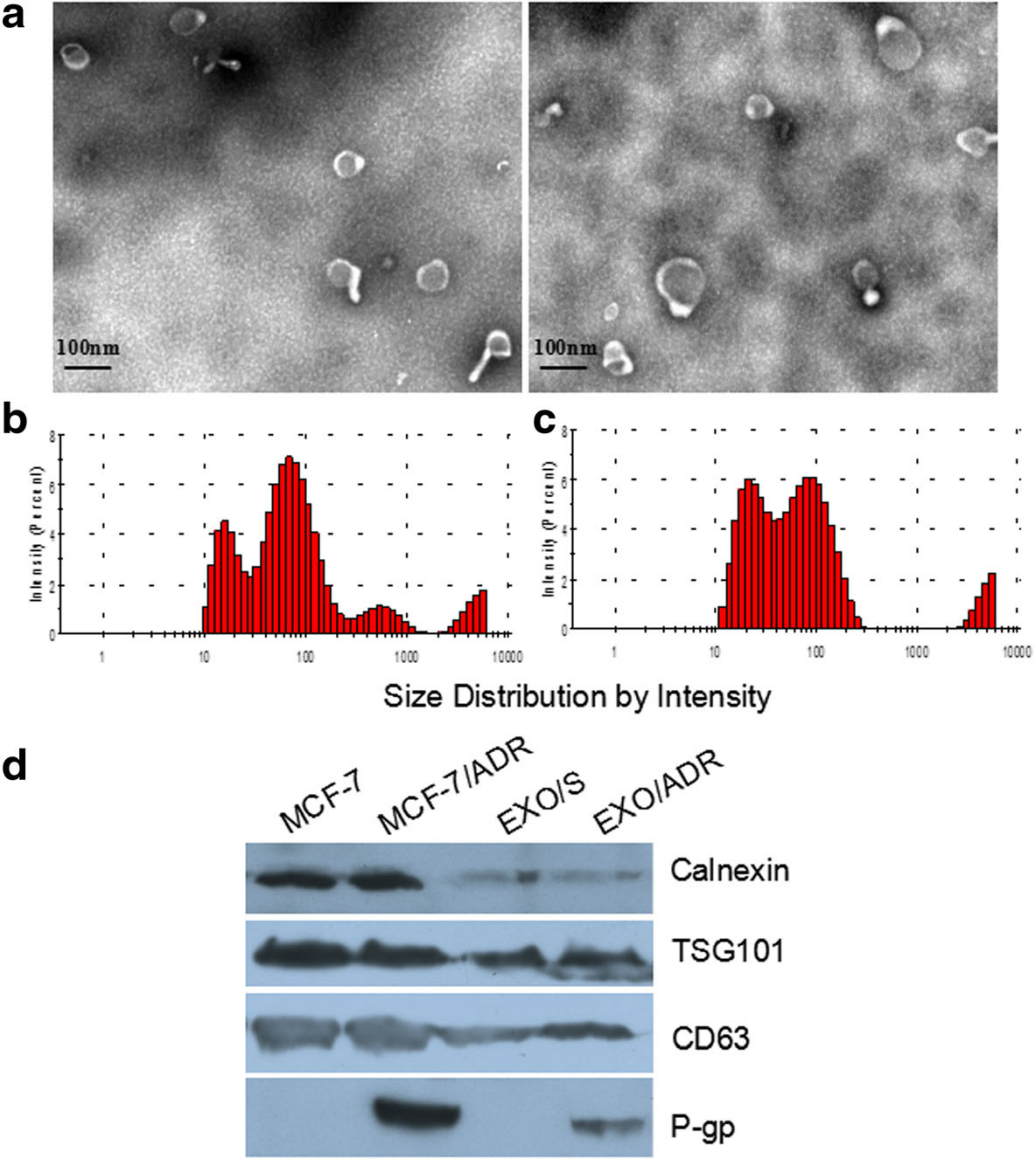
Identification and characterization of exosomes. **a** Representative transmission electron microscopy image of MCF-7 and MCF-7/ADR derived exosomes, showing a typical “saucer-like” morphology and a diameter of 30–100 nm (scale bar, 100 nm). **b** an﻿d﻿ **c** Analysis of exosome size indicated similar particle size distribution of exosomes secreted by MCF-7 and MCF-7/ADR (mean ± SD). **d ** Exosome purity as assessed by western blot analysis for the expression of the exosomal marker CD63, TSG101 and endoplasmic reticulum protein calnexin. P-gp in total cellular protein and corresponding exosomes of MCF-7 and MCF-7/ADR cells were also detected

After isolation of exosomes we characterized their from 200 mL supernatants of MCF-7/ADR, we characterized their morphology and components. Transmission electron microscopy analysis showed that the nanovesicles isolated from MCF-7 and MCF-7/ADR cells were morphologically homogeneous, ranging from 30 to 100 nm in size, with a typical round or cup shape appearance (Fig. 1a). Particle size distribution of nano-AE PBS aqueous solution was shown in Fig. 1c detected using nano-ZS90 (Malvern). About 85.9% S/exo displayed a size ranging from 17.77 to 83.36 nm and 93.9% ADR/exo ranging from 24.01 to 93.23 nm. According to the results, similar size of exosomes were secreted by MCF-7 and MCF-7/ADR cells, whereas a minority of them were >100 nm. Exosomes purity was assessed by western blot analysis. As shown in Fig. 1c, they all expressed exosome-related protein CD63 and TSG101, while calnexin was only detected in total cellular lysates and not in exosomes, indicating that our exosomes preparations are free of cellular components and debris. P-gp was expressed by both MCF-7/ADR cells and ADR/exo but is undetectable in the MCF-7 and S/exo, implicating it as involved in the acquired docetaxel-resistance. Importantly, we found that the expression pattern in the corresponding exosomes reflected that of the cells from which they were derived, further supporting the potential of resistance transfer and our suggestion that P-gp could potentially be-at least partly-involved in the newly-acquired resistance conferred by the exosomes.

### Exo/ADR transfer chemoresistance to recipient cells

**Fig. 2:**
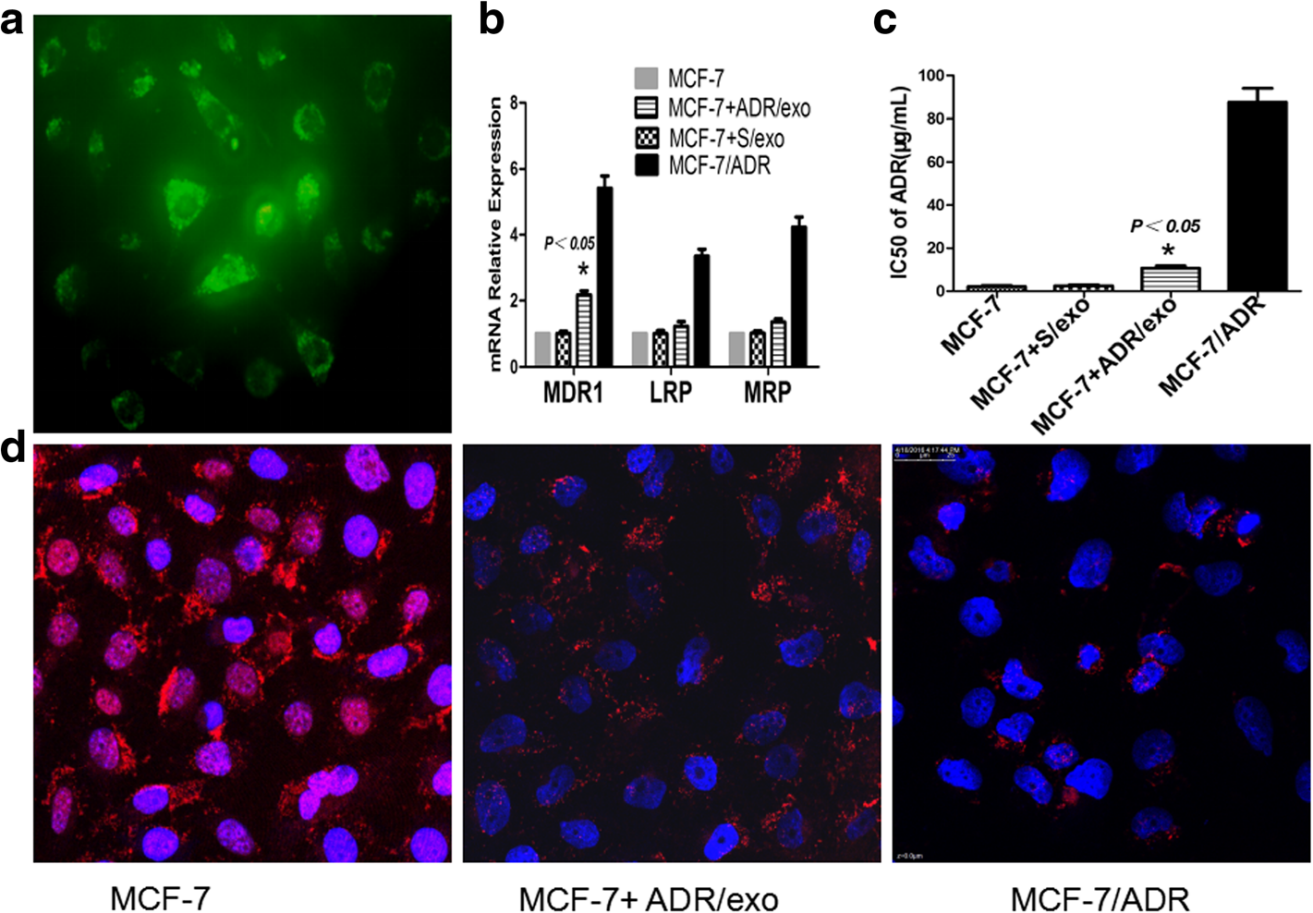
ADR/exo transfer chemoresistance to recipient cells. **a** The uptake of the fluorescently labelled Exo/ADR was evident in 90% MCF-7 cells after 12 h of incubation. No stain was revealed in the negative control condition (PBS). **b** Drug-resistance-related mRNA changes (MDR-1, MRP and LRP) in MCF-7 incubated with ADR/exo. ADR/exo induced a increase of MDR-1, MRP and LRP mRNA levels compared to MCF-7 and MCF-7 + S/exo cells, especially the MDR-1 (*p* < 0.05). **c** IC50 of adriamycin was determined by MTT. The results showed that MCF-7 cells after incubation with ADR/exo displayed 5.5 fold greater resistance to adriamycin than MCF-7 cells. MCF-7 + ADR/exo had greater resistance to adriamycin, *p* < 0.05 compared to MCF-7 cells. **d** Confocal micrographs showing adriamycin localization in MCF-7+ ADR/exo cells. Scale bars, 25 μm

To further investigate the potential of exosomes in the drug-resistance. Uptake of exosomes by MCF-7 incubated with PKH-67 labeled S/exo and ADR/exo for up to 12 h was assessed using fluorescence microscopy after extensively washing the cells to remove any extracellular exosomes. A representative image of MCF-7 cells incubated with exosomes from MCF-7/ADR cells shown in Fig. 2a. In all cases we observed 90% of MCF-7 cells containing green fluorescent exosomes. The intracellular localization of these exosomes in the MCF-7 cells were mainly in the cell membrane and cytoplasm. Then the ability of to transfer drug-resistance-related gene (MDR-1, MRP and LRP) were assessed by qRT-PCR. Incubation of MCF-7 cells with ADR/exo induced increase of MDR-1, MRP and LRP compared to untreated cells and especially the MDR-1 (*p* < 0.05). On the contrary, no substantial changes were observed when MCF-7 cells were treated with S/exo (Fig. 2b).

MTT assays were performed to assess IC50 of adriamycin. We found that MCF-7 cells after incubation with ADR/exo displayed 5.5 fold greater resistance to adriamycin than MCF-7 cells (Fig. 2c). The subcellular distribution of adriamycin was observed under a confocal laser scanning microscope. As expected, adriamycin mostly accumulated inside the nucleus in MCF-7 cells. However, in MCF-7 + ADR/exo cells, adriamycin accumulation was much lower, with most of residual adriamycin located near cell peripheral regions but not in nucleus (Fig. 2d). These findings suggested that exosomes from drug-resistant cells played an important role in cellular drug resistance. Interestingly the residual concentration of adriamycin in the MCF-7 + ADR/exo cells is not too low, while almost no adriamycin inside the nucleus. These findings suggested that exosomes from drug-resistant cells played an important role in cellular drug resistance maybe not only by transferring drug-resistant genes, but also by blocking drugs into the nucleus by active sequestration of adriamycina. In order to verify this hypothesis, we detected the adriamycin in released exosomes by UV spectrophotometer. Estimation of adriamycin was 0.872 ± 0.074 μg/ml in the adriamycin treated group but was undetectable in the control group. These results demonstrate a new mechanism of drug-resistance acquisition via exosomes.

### Psoralen reduce exosomes generation

**Fig. 3:**
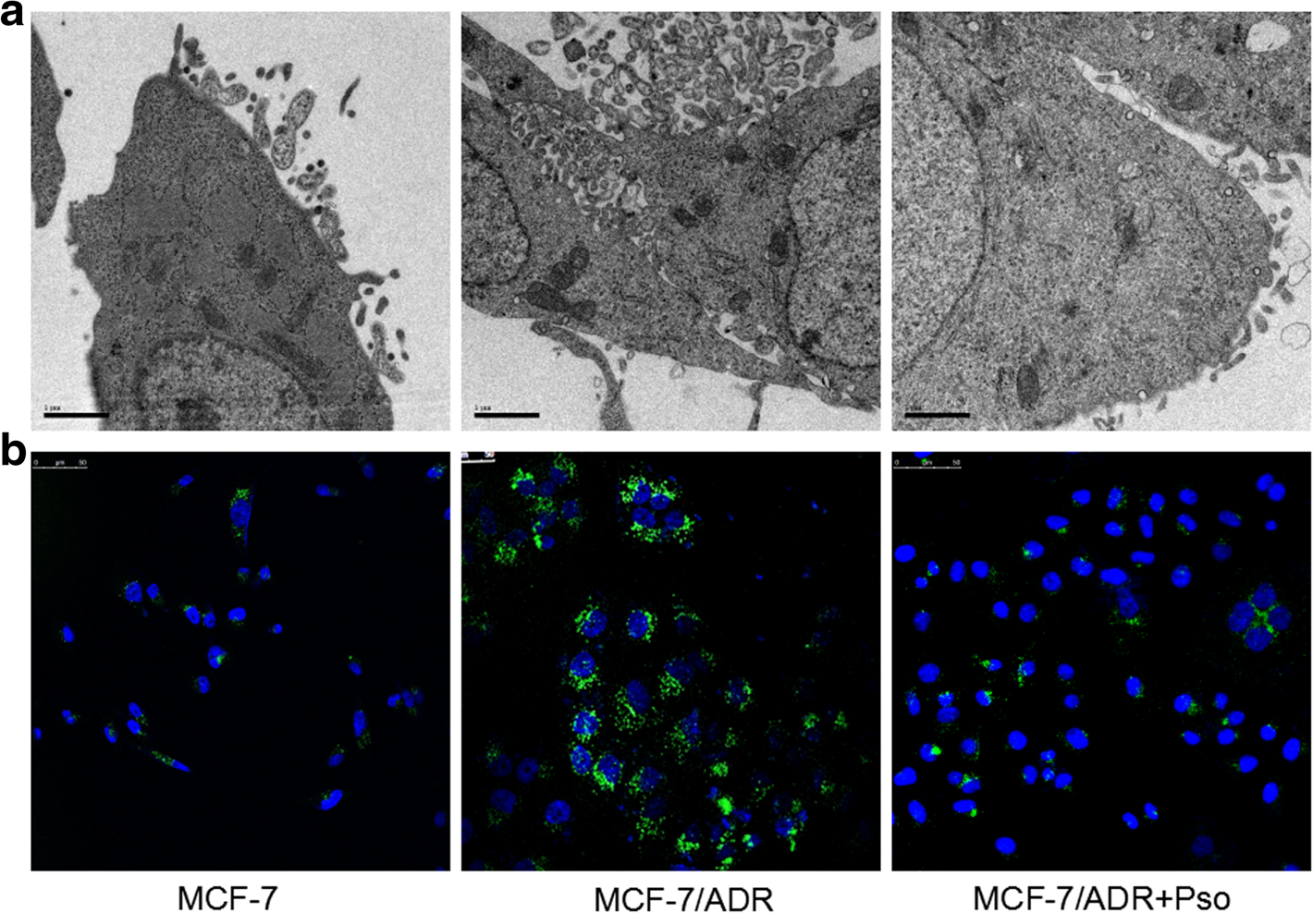
Different amounts of formation and secretion of exosomes in MCF-7, MCF-7/ADR and MCF-7/ADR + psoralen cells. **a** Transmission electron micrographs of exosomes structures (25,000×). **b** Confocal images of immunostaining for exosomes CD63 expression in MCF-7/ADR and MCF-7/ADR + psoralen cells. Scale bars, 50 μm

The effects of exosomes shed by drug-resistant cells in the dissemination of drug-resistance have been confirmed. Therefore, reducing formation and secretion of exosomes may be a novel therapeutic strategy for adjuvant cancer treatment by restoring drug sensitivity in breast cancer. Interestingly, we found a marked abundance of exosomes on the surface of MCF-7/ADR cells by transmission electron microscopy compared to MCF-7 cells, and exosomes generation significantly decreased after psoralen treatment (Fig. 3a). The vesicular structures were generated from the plasma membrane and released into the microenvironment. Immunofluorescent staining for the exosomes marker CD63 in MCF-7/ADR cells further confirmed that showed that psoralen treatment decreased the exosomes by about 40% (Fig. 3b), suggesting that the psoralen actually reduced the formation and secretion of exosomes.

### Differentially expressed genes after psoralen treatment

**Fig. 4:**
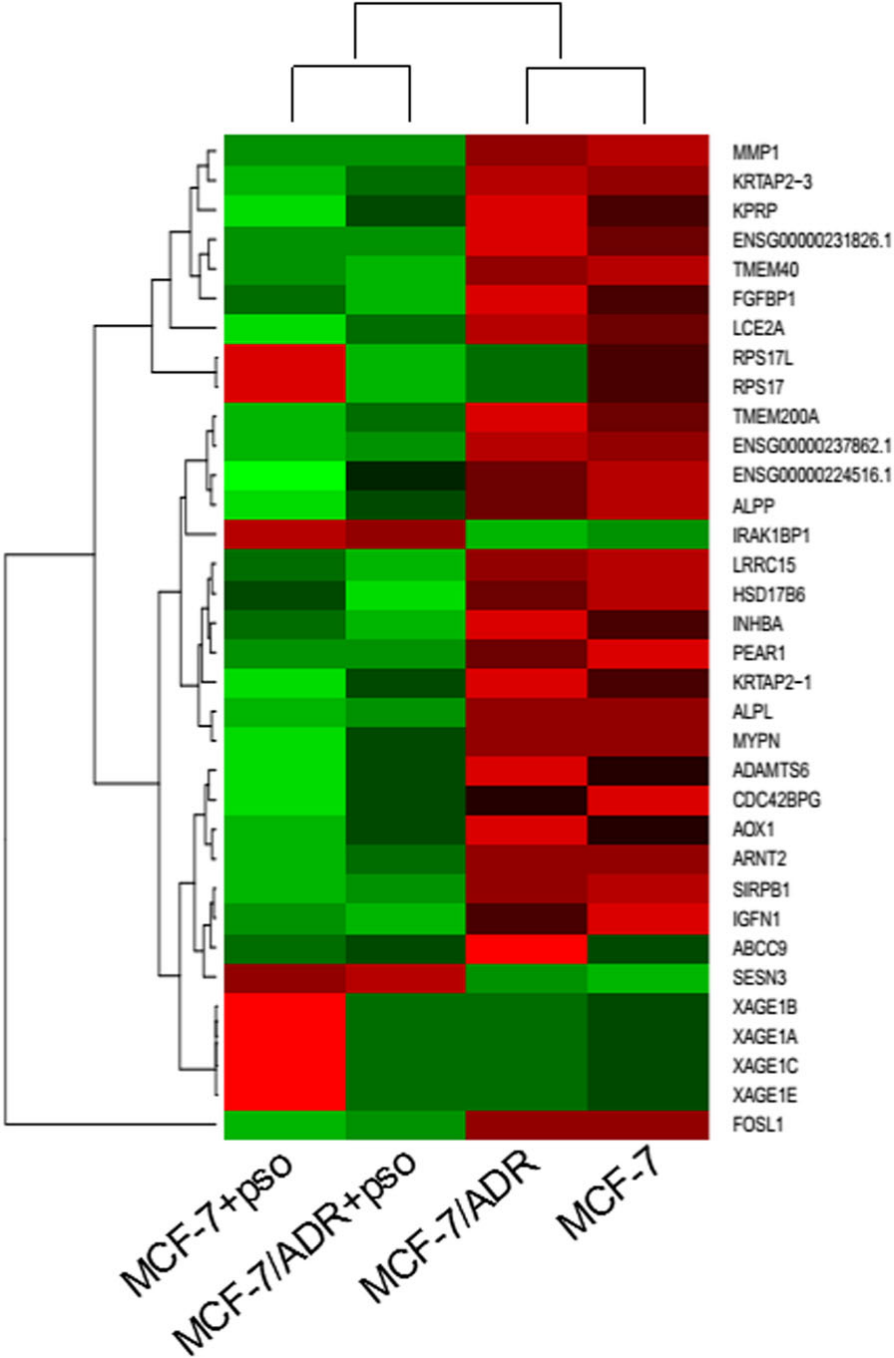
A number of genes are differentially expressed in MCF-7 and MCF-7/ADR vs. MCF-7 + psoralen and MCF-7/ADR + psoralen. Ribosomal RNA-free total RNA extracts were prepared from 24 h derived from each of 3 donors and subjected to RNA-seq analysis. A heat map was generated using the significant DE genes with a *P* value of 0.01

**Fig. 5:**
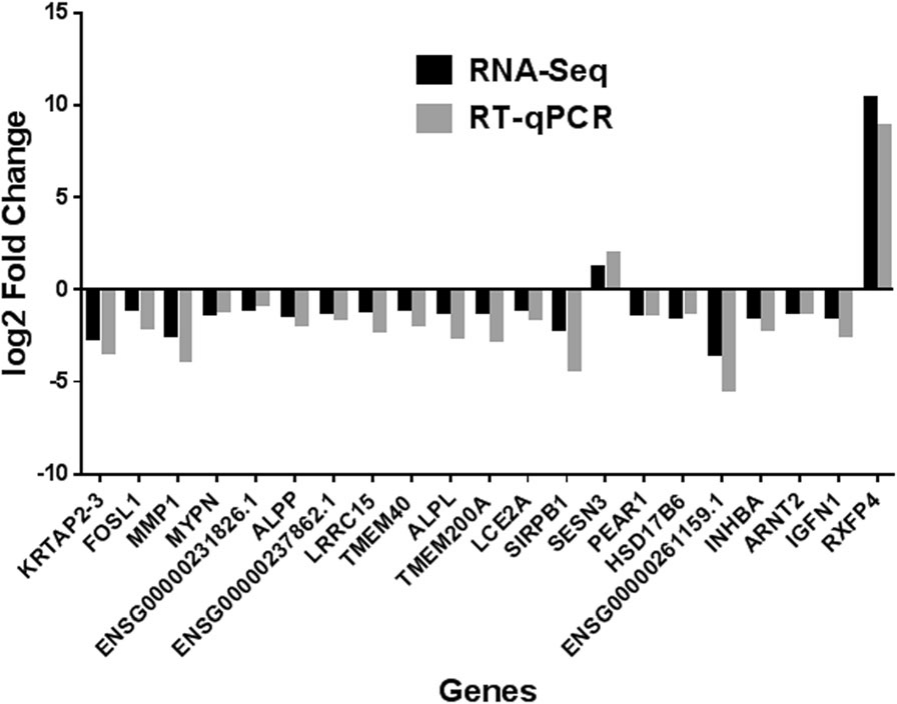
Fold Changes of DE genes (MCF-7 and MCF-7/ADR vs. MCF-7 + psoralen and MCF-7/ADR + psoralen). The barchart displays the log2 fold changes of validated candidate genes, which showed significant differences in their expression in MCF-7 and MCF-7/ADR vs. MCF-7 + psoralen and MCF-7/ADR + psoralen, respectively. Positive values indicate an up-regulation after psoralen treatment. Negative values indicate an down-regulation fter psoralen treatment. *Black bars* denote values resulting from RNA-Seq analysis. *Gray bars* denote values resulting from RT-qPCR analysis

The differential expression (DE) revealed by RNA-Seq analysis was confirmed via RT-qPCR for 21 candidate genes. The whole set consisted of 34 genes (Fig. 4). Figure 5 shows the log2 FC resulting from RNA-Seq and RT-qPCR analysis for 21 candidate genes (Table 2), respectively. The selection criterion was mainly based on literature support for association of genes with breast cancer. Among the validated candidate genes, 19 had negative log2 fold changes (FC) and 2 had positive ones. A negative log2 FC indicated significantly lower expression in psoralen treated groups compared to control groups. The genes with a positive log2 FC were significantly higher expressed in psoralen treated groups compared to control groups and might contribute to psoralen efficiency.

**Table 2:** Relative expression level of Psoralen group over that Control group

Gene symbol	Control	Psoralen	log2FoldChange	*P value*
KRTAP2-3	473.4431128	77.96785331	−2.602239769	1.55E-31
FOSL1	11296.63679	5566.561955	−1.021034857	3.49E-26
MMP1	1170.248705	212.1662542	−2.463548055	1.18E-13
MYPN	456.4454562	187.019253	−1.287255674	1.63E-10
ENSG00000231826.1	761.8035897	366.7059488	−1.054795416	7.26E-08
ALPP	320.5900834	127.107873	−1.334676407	1.22E-07
ENSG00000237862.1	247.8975043	105.2724733	−1.235615498	1.45E-07
LRRC15	313.4134599	144.8134519	−1.113871516	3.37E-07
TMEM40	398.6759476	196.9516533	−1.017375042	5.19E-07
ALPL	205.4467798	88.21207065	−1.21971673	1.31E-06
TMEM200A	714.8598445	303.7664782	−1.234697743	3.42E-06
LCE2A	455.8712616	224.9098164	−1.019279833	8.62E-06
SIRPB1	43.66423935	9.736212337	−2.165019671	1.03E-05
SESN3	73.33189937	163.1626044	1.153797628	1.94E-05
PEAR1	195.9475924	80.66338615	−1.280481972	2.38E-05
HSD17B6	83.7271271	31.44521183	−1.412854769	3.95E-05
ENSG00000261159.1	17.47612762	1.611337899	−3.439054581	6.33E-05
INHBA	92.57825197	34.35526917	−1.430141936	6.82E-05
ARNT2	115.3737968	50.98582145	−1.17814759	8.87E-05
IGFN1	87.83302938	31.6306287	−1.473441332	1.02E-04
RXFP4	0	10.4296342	Inf	1.26E-04

The table displays the results of the RNA-Seq data comparison before and after psoralen treatment. It holds the description, log2 fold change (FC) of the differentially expressed genes. Positive log2 fold changes indicate higher gene expression after psoralen treatment, while negative log2 fold changes indicate lower gene expression after psoralen treatment

SESN3 (Sestrin 3) is a Protein Coding gene. This gene encodes a member of the sestrin family of stress-induced proteins. The encoded protein reduces the levels of intracellular reactive oxygen species induced by activated Ras downstream of RAC-alpha serine/threonine-protein kinase (Akt) and FoxO transcription factor. The protein is required for normal regulation of blood glucose, insulin resistance and plays a role in lipid storage in obesity. It was overexpressed after psoralen treatment. According to GO annotation, it is associated to the p53 signaling pathway which could control the secretion of exosomes [19–21]. A p53-regulated gene product, TSAP6, was shown to enhance exosome production in cells undergoing a p53 response to stress. The p53 pathway regulates the production of exosomes into the medium and these vesicles can communicate with adjacent cells and even cells of the immune system [22].

INHBA (inhibin beta A) is a Protein Coding gene and it was down-regulated after psoralen treatment. Diseases associated with INHBA include ovary adenocarcinoma and preterm premature rupture of the membranes. Among its related pathways are PEDF Induced Signalingand Signaling pathways regulating pluripotency of stem cells. Our GO enrichment analysis and the KEGG pathway analysis showed that it is associated to the TGF-β signalling which contributes to dysregulation of sphingolipid metabolism [23]. Sphingomyelin and its metabolic products, particularly ceramide and sphingosine 1-phosphate, have a major role in exosomes biogenesis and microvesicle shedding [24].

HSD17B6 (Hydroxysteroid (17-Beta) Dehydrogenase 6) is a Protein Coding gene and it was down-regulated after psoralen treatment. Diseases associated with HSD17B6 include ovarian endometrioid stromal sarcoma and ovary sarcoma. Among its related pathways areMetabolism and Metabolism of xenobiotics by cytochrome P450. GO annotations related to this gene include oxidoreductase activity and retinol dehydrogenase activity. The KEGG pathway analysis showed that it is associated to the steroid hormone biosynthesis and retinol metabolism. Both of them are speculated to participate in sphingolipids metabolism such as ceramide, sphingosine, sphingosine-1-phosphate and sphingomyelin, mediating exosomes biogenesis and microvesicles shedding.

Another down-regulated gene after psoralen treatment was MMP1 (Matrix Metallopeptidase 1) as a protein coding gene. Diseases associated with MMP1 include epidermolysis bullosa dystrophica, ar and pulmonary disease, chronic obstructive. Among its related pathways areBladder cancer and Pathways in cancer. GO annotations related to this gene include calcium ion binding and metallopeptidase activity. The KEGG pathway analysis showed that it is associated to the PPAR signaling pathway. PPAR signaling pathway regulates the synthesis of ceramide levels, and ceramide is an important regulatory molecule in exosomes secretion [25, 26].

## Discussion

Chemotherapy is a primary strategy to treat breast cancer in patients, the MDR, both intrinsic and acquired, is still a major concern regarding the clinical management of OS patients which severely increasing mortality in patients undergoing chemotherapy. Previously, we showed that psoralen significantly reversed MDR in human breast cancer MCF-7/ADR cells but didn’t reduce MDR-related genes expression [27]. Our study showed that exosomes shed by drug-resistant cells contribute to the dissemination of MDR by transferring their cargo to drug-sensitive cells. The cargo of the drug-resistant exosomes may be selectively packaged and may include drug-efflux pumps [28]. The drug-efflux pumps transferred by exosomes to drug-sensitive cells are functional in the recipient cells. Drug-efflux pumps carried by exosomes may be responsible for the sequestration of drugs in those exosomes as we can see that there were adriamycin detected in released ADR/exo.

In this study, cells incubation with exosomes derived from MCF-7/ADR cells increased adriamycin resistance in MCF-7 cells with a reversal of 5.5 fold and the uptake ratio reached up to 90%. We also elucidated that the release of exosomes by MCF-7/ADR cells enables drug resistance not only by transferring exosomes carrying MDR-1 mRNA and its product P-gp, also by removing anticancer drugs that have entered the cells. As we’ve seen in other studies, the different reversal fold changes may be associated with different incubation time and different amount of exosomes [29]. Exosomes may also negatively impact chemotherapy treatment by shuttling out chemotherapeutic agents from target cancer cells. Cisplatin and doxorubicin were found in cancer cell-derived exosomes in a post treatment setting [30, 31]. We have confirmed the role of exosomes in formation and transmission of drug resistance in breast cancer.

In previous studies, psoralen have been found to lower multidrug resistance, including that against adriamycin in breast cancer cells. However, these previous studies did not invoke exosomes-associated secretion of drug-resistant genes and drug encapsulation as the potential mechanisms. In this study, we have proved that psoralen could influence the formation and secretion of exosomes and induce the reduction of resistance transmission via exosomes which provides a basis for the rational design of new treatment strategies that target and inhibit the exosomes-mediated transfer of MDR during treatment. We applied successfully the RNA-Seq method to detect differentially expressed genes after psoralen treatment. A high proportion of validated genes by RT-qPCR confirmed our results. Based on literature research, we elucidated the role of these candidate genes in influencing exosomes formation and secretion. A future deeper analysis of single candidates could reveal more detailed information about mechanisms leading to exosomes formation and secretion.

## Conclusions

Our findings contributed to a better understanding of the role of exosomes in drug-resistance acquisition and investigated the molecular mechanism of psoralen inhibition of exosomes released by MCF-7/ADR. This provides a basis for the rational design of new treatment strategies that target and inhibit the exosomes-mediated MDR during treatment.

## Abbreviations

ADR: Adriamycin
ADR/exo: Exosomes from MCF-7/ADR cells
GO pathway analysis: Gene ontology pathway analysis
KEGG pathway analysis: Kyoto Encyclopedia of Genes and Genomes pathway analysis
MDR: Multidrug resistance
qRT-PCR: Real-time quantitative PCR
S/exo: Exosomes from MCF-7 cells
